# Emotion regulation difficulties in anorexia nervosa: associations with improvements in eating psychopathology

**DOI:** 10.1186/s40337-016-0108-0

**Published:** 2016-05-18

**Authors:** Marsha Rowsell, Danielle E. MacDonald, Jacqueline C. Carter

**Affiliations:** Department of Psychology, Memorial University of Newfoundland, St. John’s, NL A1B 3X9 Canada; Department of Psychology, Ryerson University, Toronto, Ontario M5G 2C4 Canada; Department of Psychiatry, University Health Network, Toronto, Ontario M5G 2C4 Canada

**Keywords:** Anorexia nervosa, Emotion regulation

## Abstract

**Background:**

Difficulties with emotion regulation have been established as a core deficit in anorexia nervosa (AN). However, limited research has evaluated whether weight gain is associated with improvements in emotion regulation difficulties in AN and whether improvements in emotion regulation are associated with reductions in eating disorder psychopathology. The aims of this study were threefold: 1) to examine the nature and extent of emotion regulation difficulties in AN; 2) to determine whether these difficulties improved during intensive treatment for the eating disorder; and 3) to study whether improvements in emotion regulation were associated with improvements in eating disorder psychopathology.

**Method:**

The participants were 108 patients who met DSM-IV-TR criteria for AN and were admitted to a specialized intensive treatment program. Self-report measures of eating disorder symptoms and difficulties with emotion regulation were administered at admission to and discharge from the program.

**Results:**

Patients with the binge-purge subtype of AN reported greater difficulties with impulse control when upset and more limited access to emotion regulation strategies when experiencing negative emotions than those with the restricting subtype. Among those who completed treatment and became weight restored, improvements in emotion regulation difficulties were observed. Greater pre-to-post treatment improvements in emotional clarity and engagement in goal directed behaviours when upset were associated with greater reductions in eating disorder psychopathology during treatment.

**Conclusions:**

These findings add to growing evidence suggesting that eating disorder symptoms may be related to emotion regulation difficulties in AN and that integrating strategies to address emotion regulation deficits may be important to improving treatment outcome in AN.

## Background

In her seminal writings, Bruch [1, 2] asserted that problems with emotion regulation, particularly difficulties differentiating and describing emotions, are a core deficit in anorexia nervosa (AN). Recently, a growing body of theoretical and empirical evidence suggests that emotion regulation deficits may play a key role in both the development and maintenance of AN [3–7]. Indeed, several authors have theorized that AN *is* a disorder of emotion regulation and that the symptoms of AN, such as dietary restriction, excessive exercise, and binge/purge behaviors, represent maladaptive attempts to regulate aversive emotional states [8–10].

In a recent review article, Lavender and colleagues [11] examined the research evidence for applying Gratz and Roemer’s [12] multidimensional model of emotion dysregulation in conceptualizing emotion regulation difficulties in AN. This review uncovered the following findings: considerable evidence to support the application of this model in AN including evidence of broad deficits in adaptive emotion regulation skills in AN; some evidence of impulse control and distress tolerance difficulties in AN; substantial evidence of emotion awareness deficits in AN; and some evidence of emotional avoidance in AN.

Indeed, a number of studies have found that difficulties with emotion regulation are associated with eating disorder psychopathology [7, 13]. High levels of negative emotionality have been shown to prospectively increase the risk of eating pathology [14, 15] and, in a recent meta-analysis, lack of adaptive emotion regulation strategies – particularly deficits in problem-solving skills, and greater use of avoidance, rumination and suppression – was associated with greater eating disorder psychopathology [16]. Further, there is evidence that characteristic anxious avoidance, both of emotions and of interpersonal situations that may trigger emotional experiences in AN, often predates the illness and persists after recovery [7, 17, 18].

Several recent studies have found evidence that emotion regulation difficulties may also play a role in the maintenance of AN. It has been consistently found that individuals with AN report greater impairments in emotion regulation compared to controls [19]. Specifically, AN has been shown to be associated with high vulnerability to dysregulated emotions including impaired abilities to experience and differentiate emotions, as well as difficulties with the attenuation and modulation of negative emotional states [19–21]. Individuals with AN have also been found to report higher levels of alexithymia (i.e., difficulty identifying and describing emotions) compared to healthy controls [22, 23]. Further, some research has shown that individuals with AN have greater levels of alexithymia than individuals with other eating disorders such as bulimia nervosa and binge eating disorder [23], suggesting a specific emotion-related deficit in AN rather than a general impairment related to eating disorders more broadly. In addition, several studies have found evidence of distress intolerance, that is, the inability to accept and withstand negative emotional experiences, particularly among those with the restricting subtype of AN [4, 17]. As observed by Lynch and colleagues [24], emotional overcontrol has been linked with social withdrawal, cognitive rigidity, reward insensitivity, strong needs for structure and symmetry, and clinical perfectionism – traits which have been shown to be common among individuals with AN, and which may function to perpetuate the disorder [6, 25, 26].

Studies that have examined differences in emotion regulation deficits between the two subtypes of AN have produced mixed results. In the restricting subtype of AN (AN-R), emotion regulation deficits tend to be characterized by emotional overcontrol, such as emotional inhibition and lack of emotion expression [27]. Certain symptoms of AN-R, such as dietary restriction or excessive exercise, may be conceptualized as maladaptive attempts to avoid emotional arousal and inhibit emotional expression. In support of this theory, Wildes and colleagues [10] found that emotion avoidance partially mediated the relationship between depressive symptoms and eating disorder psychopathology in AN, suggesting that eating disorder symptoms may function to regulate negative mood states. In contrast, the findings of another recent study did not support an emotional avoidance model of AN [28].

In the binge-purge subtype of AN (AN-BP), difficulties with emotion regulation tend to be characterized by impulsivity and difficulties inhibiting maladaptive behavior when experiencing negative emotions [21, 29]. Further, negative emotions have been shown to be a strong proximal trigger for binge eating and purging episodes in AN-BP [10]. Racine and Wildes [30] examined whether specific aspects of the psychopathology of acute AN were uniquely associated with particular types of emotion regulation difficulties. They found that, impulse control difficulties predicted unique variance in both binge eating and purging behaviors, over and above the effects of duration of illness, and depression and anxiety symptoms, respectively. Additionally, limited emotional awareness uniquely predicted the severity of eating disorder cognitions over and above duration of illness, and depression and anxiety symptoms. In another study of inpatients with AN, only one difference was found between patients with AN-R and AN-BP: AN-BP patients had higher impulsivity scores compared to those with AN-R [31]. Further research is needed to clarify differences between the two subtypes of AN with respect to emotion regulation deficits since this may have important potential treatment implications.

The relationship between low body weight in acute AN and emotion regulation difficulties remains unclear. Brockmeyer and colleagues [20] found that lower body mass index was associated with fewer emotion regulation difficulties in a sample of patients with acute AN, suggesting that low weight may function as a persistent but maladaptive means of over-regulating negative affect during the acute illness period. That is, low body weight may lower the frequency or intensity of negative emotions thereby functioning as a form of emotional avoidance. However, given the well-established negative impact of starvation on psychological functioning [32], it is also possible that weight restoration may improve emotion regulation abilities in AN. Only two studies to date have examined whether weight restoration is associated with improvements in emotion regulation in AN. Haynos and colleagues [31] found that difficulties with emotion regulation did not improve with weight restoration through specialized inpatient treatment although significant improvements in both general psychological distress and specific eating disorder psychopathology were found. Conversely, Ben-Porath, Federici, Wisniewski, and Warren [33] found that problems with emotion regulation improved with weight restoration in AN through a specialized day treatment program that included two hours of dialectical behavioral therapy (DBT) skills training per week. However, because the treatment program in this study included DBT skills training, which is an empirically supported intervention for problems with emotion regulation, it is unknown whether the improvements in emotion regulation were related to skills training or to weight restoration. In addition, while Haynos and colleagues [31] studied a sample of individuals who were all diagnosed with AN, Ben-Porath and colleagues [33] recruited a mixed sample of individuals diagnosed with either AN (~35 %) or BN (~65 %). As such, participants in Ben-Porath study had a greater mean admission BMI than participants in Haynos and colleagues’ [31] study, and not all participants in the former study were underweight, making it difficult to compare findings across the studies. Overall, however, it is clear that there is a lack of research with respect to the relationship between weight restoration, improvements eating disorder psychopathology, and changes in emotion regulation difficulties. As such, additional research on this topic is necessary in order to elucidate and clarify the nature of these relationships. In support of this, recently psychological science has shown a renewed interest in the area of study replication [34]. Indeed, researchers have coined replication “the cornerstone of science” [35], highlighting its importance in determining whether any given finding is a false positive or the result of sampling error or non-generalizable sample specific features [34, 35]. Thus, the current study aimed to replicate and extend research on difficulties with emotion regulation in AN.

### Study aims and hypotheses

This study had three aims. The first aim was to replicate previous research by comparing patients with AN-R and AN-BP in terms of the nature and extent of difficulties with emotion regulation at the time of admission to intensive treatment using the Difficulties in Emotion Regulation Scale (DERS) which is based on Gratz and Roemer’s multidimensional model of emotion regulation and dysregulation [12]. Given previous findings indicating greater impulsivity and more difficulties inhibiting maladaptive behaviors when experiencing negative emotions in AN-BP, it was hypothesized that those with AN-BP would report greater difficulties with emotion regulation overall, as well as greater difficulties with impulse control in particular. Because emotional overcontrol and associated traits have been observed to be common in AN-R, it was hypothesized that patients with the AN-R subtype would have greater difficulties with lack of emotion awareness and non-acceptance of emotions than those with AN-BP.

The second aim of this study was to replicate and extend the work of Haynos and colleagues [31] by examining whether the level of various difficulties with emotion regulation changed during intensive eating disorder treatment among those who successfully completed the program. Given that our program involves extensive psychological treatment including a group focused on enhancing emotion regulation skills, it was hypothesized that, even after controlling for the effects of weight gain across treatment, the overall level of difficulties with emotion regulation would improve from pre-treatment to post-treatment. A secondary exploratory aim was to examine whether there were improvements in the specific facets of emotion regulation difficulties measured by the DERS: awareness and clarity of emotions; emotion acceptance; ability to engage in goal-directed behaviors while experiencing negative emotions; tendency to engage in impulsive behaviors when upset; and access to emotion regulation strategies.

The final aim of this study was to examine whether changes in the various facets of emotion regulation difficulties were associated with changes in eating disorder psychopathology during treatment, after controlling for baseline level of negative affect and amount of weight gain achieved during treatment. Given previous findings suggesting that specific aspects of the psychopathology of AN were uniquely associated with particular types of emotion regulation difficulties, it was hypothesized that greater improvements in the various facets of emotion regulation would be associated with greater improvements in eating disorder psychopathology, even after controlling for the effects of baseline negative affect and weight gain during treatment.

## Method

### Participants and treatment

The participants were 108 consecutive patients who met *Diagnostic and Statistical Manual of Mental Disorders*, *Fourth Edition, Text Revision* (DSM-IV-TR) criteria for AN based on assessment using the Eating Disorder Examination (EDE) interview [36, 37]. All participants had a BMI ≤ 17.5 at admission to the program. See Table 1 for demographic and clinical characteristics of the sample. Participants were admitted to the hybrid inpatient/day treatment unit of the Eating Disorders Program at a large general hospital between 2010 and 2014. This program is a specialized hospital-based program operated by an interdisciplinary team consisting of psychiatrists, psychologists, nurses, dieticians, social workers, and occupational therapists. The program goals include medical stabilization, weight restoration, normalized eating through staff-supported meals, as well as eradication of binge eating, purging, and excessive exercise (citation omitted for blind review). Although the underlying orientation of the program is cognitive-behavioural, patients attend a variety of groups including interpersonal therapy, and DBT skills training. Once patients reach a BMI of approximately 18, they are transferred from inpatient to day attendance. For the present study, post-treatment BMI and weight were available for the 72 patients who completed the inpatient/day program and achieved a BMI ≥ 19.5. Questionnaire data were available for 53 of the 72 participants who completed treatment and achieved a BMI of at least 19.5. This study was approved by the UHN Research Ethics Board and all participants provided written informed consent to participate.

**Table 1:** Demographic and clinical characteristics of the sample (*N* = 108)

Characteristic	Mean (SD) or %
Female	96.3 %
Age	29.9 (10.9)
Ethnicity	
Caucasian	88.1 %
Asian	3.0 %
Other^a^	10.9 %
Marital Status	
Single	72.6 %
Married/Common-Law	22.6 %
Separated/Divorced	4.7 %
Employment Status	
Student	30.4 %
Employed	48.1 %
Unemployed	21.6 %
AN Subtype	
AN-R	41.5 %
AN-BP	58.5 %
Pre-treatment BMI	14.9 (1.4)
Binge episodes/month Pre-Treatment (AN-BP only)	12.3 (23.1)
Vomit episodes/month Pre-Treatment (AN-BP only)	37.9 (54.9)
Laxative episodes/month Pre-Treatment (AN-BP only)	10.6 (16.5)
Duration of Illness (Years)	10.6 (9.6)
Age of Onset	19.4 (7.7)
Length of Treatment (Weeks)	14.4 (7.1)
Weight Gain During Treatment (kg)	11.5 (6.1)

^a^Other reported ethnicities include Black, West Indian, East Indian, and Hispanic

### Assessment measures

#### Eating disorder psychopathology

The 36-item Eating Disorder Examination–Questionnaire (EDE-Q) was used as a measure of eating disorder psychopathology [38]. This questionnaire is based directly on the EDE interview and is comprised of four subscales (Shape Concern, Weight Concern, Eating Concern, and Dietary Restraint) that can be combined into one Global Score of eating disorder psychopathology ranging from 0 (low) to 6 (high). The EDE-Q has demonstrated good test-retest reliability and strong internal consistency [39]. The Cronbach’s alpha for the Global Score in our sample was α = .91 and α = .93 at pre- and post-treatment, respectively. In addition, the EDE-Q contains items assessing the frequency of eating disorder behaviors including binge eating and compensatory behaviors.

#### Emotion regulation difficulties

The Difficulties in Emotion Regulation Scale (DERS) is a multidimensional 36-item self-report questionnaire that measures aspects of emotion dysregulation [12]. The DERS provides a total score and six subscale scores including: 1) lack of emotional clarity (Clarity); 2) lack of emotional awareness (Awareness); 3) non-acceptance of emotional responses (Non-acceptance); 4) Impulse control difficulties (Impulse); 5) difficulties engaging in goal directed behavior in the presence of negative emotions (Goals); and 6) limited access to emotion regulation strategies (Strategies). Each item is rated on a 5-point scale ranging from “almost never (0–10 %) to “almost always (91–100 %)”. Reliability and validity of this instrument has been established [9]. In the current sample, Cronbach’s alpha for the total scale was α = .96, and for each of the six subscales it was α = .86 (Clarity), α = .85 (Awareness), α = .94 (Non-Acceptance), α = .90 (Impulse), α = .88 (Goals), and α = .92 (Strategies). At post-treatment, Cronbach’s alpha for the total scale was α = .95 and for each of the six subscales it was α = .91, α = .89, α = .93, α = .85, α = .91, and α = .92 respectively.

### Procedure

Upon being admitted to the program, participants were assessed using the diagnostic items of the EDE interview [35] by a trained interviewer, and objective weight and height were measured. The validity and reliability of the EDE, including interrater reliability, have been well established and were not examined as part of the current study [40].

This information was used by the program psychologist or psychiatrist for diagnosis and subtyping. Prior to beginning the program, all participants completed the EDE-Q and the DERS. The treatment program was delivered as usual, and the EDE-Q and DERS were re-administered at the time of discharge.

### Statistical analyses

To determine whether there were any baseline differences between treatment completers and non-completers, these two groups were compared on several pre-treatment variables. Specifically, a series of independent samples *t*-tests were conducted to determine whether treatment completers and non-completers differed on: (a) emotion regulation difficulties at admission; (b) frequency of binge eating episodes, self-induced vomiting episodes, and episodes of laxative misuse at admission; and (c) BMI at admission. To reduce the distributional skewness of behavioral episodes (binge, vomit, and laxative), univariate outliers (i.e., z = +/− 3.29) were identified and replaced with the next highest value in the distribution that was not an outlier [41]. This method corrected distributional problems. Lastly, a chi square test was conducted to examine whether completers and non-completers differed in terms of frequency of AN-subtypes.

In order to address our first aim, an analysis of variance (ANOVA) was conducted to compare the two AN subtypes (AN-R and AN-BP) on the total DERS score. Additionally, a multivariate analysis of variance (MANOVA) was conducted to compare the AN-R and AN-BP subtypes on the six DERS subscales. The overall multivariate effect, as well as the individual univariate effects, were examined.

Secondly, a mixed MANOVA was conducted for the subset of participants who completed treatment (*N* = 53) to evaluate: (a) whether difficulties in emotion regulation would significantly improve from pre-treatment to post-treatment; and (b) potential subtype by time interaction effects. Pre-to-post treatment changes in DERS subscale scores were calculated as change scores. Subsequently, a second mixed MANOVA was conducted to evaluate whether difficulties in emotion regulation would significantly improve from pre-to-post-treatment when controlling for the effect of weight gain during treatment. In this analysis, pre-to-post-treatment weight gain was included as a covariate.

Finally, the hypothesis that change in emotion regulation difficulties would be associated with change in eating disorder psychopathology was investigated using multiple regression analysis. For this analysis, the criterion variable was pre-to-post-treatment change in eating disorder psychopathology as measured by the Global EDE-Q score, and the predictor variables were change in each of the DERS subscales pre-to-post-treatment. Initially we had planned to include baseline anxiety and depression symptoms, as measured by the Brief Symptom Inventory (BSI) [42], and weight gain during treatment, calculated as a change score, as covariates in this regression analysis. However, because we found that change in EDE-Q Global score was not significantly related to the level of anxiety and depression symptoms at pre-treatment (*r* = .05, *p* = .72) or to weight gain during treatment (*r* = .11, *p* = .43), the analyses were conducted without controlling for these variables. First, a series of univariate regression analyses were conducted to determine whether improvement in each facet of emotion regulation was associated with improvement in eating disorder psychopathology across treatment. Based on the results of these separate univariate regressions, a stepwise multiple regression analysis was then conducted with all six DERS subscales included as potential predictors. Based on the results of this model, a second multiple regression analysis was performed, including only the predictors that provided a significant contribution to the initial stepwise model, entered together at the same step.

## Results

### Comparisons between completers and Non-completers

With respect to potential differences between program completers and non-completers, there were no statistically significant differences between these two groups on baseline DERS Goals, *t*(106) = .07, *p* = .94; Impulse, *t*(106) = 1.38, *p* = .17; Awareness, *t*(106) = .07, *p* = .94; Strategies, *t*(106) = 1.61, *p* = .11; Clarity, *t*(106) = 1.35, *p* = .18; Non-acceptance, *t*(106) = .62, *p* = .54; and total, *t*(106) = 1.20, *p* = .23 scores. Mean subscale and total DERS scores were comparable, though somewhat higher, than previous reports of AN samples [e.g., 30, 31]. Furthermore, completers and non-completers did not significantly differ in terms of baseline frequency of binge eating, *t*(59) = .29, *p* = .78, or self-induced vomiting, *t*(59) = 1.15, *p* = .26. However, non-completers reported significantly more episodes of laxative misuse at baseline than completers, *t*(59) = 2.59, *p* = .01. Moreover, completers and non-completers did not differ significantly in terms of AN subtype, *χ*2 (1) = 3.60, *p* = .06. Finally, completers and non-completers did not significantly differ in term of baseline BMI, *t*(106) = −1.87, *p* = .07. Overall, these findings suggest that there were few meaningful pre-existing differences between completers and non-completers. See Table 2 for means and standard deviations of pre-treatment variables for completers versus noncompleters.

**Table 2:** Means and standard deviations of pre-treatment variables for completers (*N* = 72) and non-completers (*N* = 36)

	Mean (SD)	
Variable	Completers	Non-Completers
DERS-Goals	18.99 (4.75)	19.06 (5.08)
DERS-Impulse	16.60 (6.64)	18.52 (7.20)
DERS-Awareness	20.97 (5.23)	21.06 (6.20)
DERS-Strategies	25.53 (8.63)	28.44 (9.31)
DERS-Clarity	16.40 (4.51)	17.72 (5.29)
DERS Non-Acceptance	20.01 (7.06)	20.94 (7.98)
DERS-Total	118.50 (26.42)	125.75 (34.91)
Binge episodes/month (AN-BP)	13.86 (30.35)	25.46 (63.79)
Vomit episodes/month (AN-BP)	33.58 (55.86)	54.58 (82.82)
Laxative episodes/month (AN-BP)	7.11 (16.00)	24.80 (50.61)
BMI	15.11 (1.33)	14.56 (1.54)

*Note*. DERS = difficulties in emotion regulation scale

### Subtype comparisons

In accordance with our first hypothesis, there was a significant difference with respect to baseline emotion regulation difficulties, as measured by the DERS total subscale, between AN-R and AN-BP subtypes, *F*(1, 104) = 9.28, *p* = .003, R^2 =^ .08. Additionally, significant differences in baseline emotion regulation difficulties, as measured by DERS subscales, were yielded between the AN-R and AN-BP subtypes, Wilks’s λ = .83, *F*(6, 99) = 3.38, *p* = .004. Examination of the univariate effects indicated that the AN-BP subtype exhibited significantly greater impairments with respect to the Impulse subscale, *F*(1,104) = 16.63, *p* < .001, R^2^ = .14, and Strategies subscale, F(1,104) = 10.48, *p* = .002, R^2^ = .09. This indicates that, when experiencing negative emotions, patients with the AN-BP subtype reported greater difficulties with impulse control and more limited access to emotion regulation strategies than those with the AN-R subtype. There were no significant group differences with respect to the remaining DERS subscales, *p*s > .05. See Table 3 for these DERS subscale scores by subtype, as well as DERS total scores (which were not included in the model but are provided in the table for descriptive purposes).

**Table 3:** Means and standard deviations for the DERS subscales at pre-treatment for AN-BP and AN-R subtypes

	Mean (SD)		*p*
Measure	AN-BP (*n = 62*)	AN-R (*n = 44*)	
DERS-Goals	19.52 (4.26)	18.02 (5.43)	.12
DERS-Impulse	19.32 (6.42)	14.18 (6.35)	< .001
DERS-Awareness	21.42 (5.19)	20.18 (6.01)	.25
DERS-Strategies	28.63 (7.46)	23.18 (9.87)	.002
DERS-Clarity	17.23 (4.18)	16.02 (5.45)	.24
DERS-Non-acceptance	21.31 (7.23)	18.55 (7.24)	.06
DERS-Total	127.42 (25.53)	110.29 (31.66)	.003

*Note*. DERS = difficulties in emotion regulation scale

*Note*. DERS total scores were not included in the multivariate model but are provided in the table for descriptive purposes. The *p* value for the total score comes from an independent samples *t* test run for descriptive purposes only

### Pre-to-post treatment analyses

In accordance with our second hypothesis, the results from our first mixed MANOVA showed an overall multivariate effect of time on participants’ emotion regulation difficulties from pre-treatment to post-treatment, Wilks’s λ = .52, *F*(6, 46) = 7.01, *p* < .001. Given that the multivariate effect of time on emotion regulation difficulties was statistically significant, univariate effects were examined. The univariate effects for time indicated significant differences for each of the DERS subscale change scores from pre-treatment to post-treatment, indicating that across subtypes, participants made significant changes over time on all six of the DERS subscales: (1) Goals, *F*(1, 51) = 4.94, *p* = .03; (2) Impulse, *F*(1, 51) = 26.50, *p* < .001; (3) Awareness, *F*(1, 51) = 16.18, *p* < .001; (4) Strategies, *F*(1, 51) = 10.06, *p* < .01; (5) Clarity, *F*(1, 51) = 15.51, *p* < .001; and (6) Non-Acceptance, *F*(1, 51) = 5.51, *p* = .02. Additionally, the multivariate interaction effect of subtype and time was statistically significant, Wilks’s λ = .75, *F*(6,46) = 2.52, *p* = .03, indicating that there were significant differences between AN-R and AN-BP in terms of degree of improvement in emotion regulation during treatment. The univariate effects indicated a significant group by time interaction on the Impulse subscale, *F*(1, 51) = 13.71, *p* = .001. Examination of the means indicated that AN-BP improved more during treatment than AN-R, with respect to the ability to inhibit impulsive behaviors when experiencing negative emotions. See Table 4 for these DERS subscale change scores by subtype, as well as DERS total change scores (which were not included in the model but are provided in the table for descriptive purposes).

**Table 4:** Overall means and standard deviations of DERS subscales from Pre- to post-treatment (*n* = 53)

	Mean (SD)		*p*
Measure	Pre-Treatment	Post-Treatment	
DERS-Goals	18.26 (4.78)	16.87 (5.31)	.03
DERS-Impulse	16.23 (6.64)	12.26 (4.95)	< .001
DERS-Awareness	20.51 (5.49)	17.43 (5.15)	< .001
DERS-Strategies	24.74 (8.80)	21.04 (8.00)	.003
DERS-Clarity	16.26 (4.24)	13.19 (4.72)	< .001
DERS-Non-acceptance	19.57 (6.99)	17.53 (6.79)	.02
DERS-Total	115.57 (25.76)	98.32 (25.75)	< .001

*Note*. DERS = difficulties in emotion regulation scale

*Note*. DERS total scores were not included in the multivariate model but are provided in the table for descriptive purposes. The *p* value for the total score comes from an independent samples *t* test run for descriptive purposes only

When pre-to-post treatment weight gain was included as a covariate in our second mixed MANOVA, there was no longer a multivariate effect of time on emotion regulation difficulties from pre-treatment to post-treatment, Wilks’s λ = .85, *F*(6, 45) = 1.32, *p* **=** .27, indicating that across subtypes, patients did not exhibit overall improvements in emotion regulation after controlling for the effects of weight gain. However, the multivariate interaction effect of subtype by time remained statistically significant after controlling for the effects of weight gain, Wilks’s λ = .75, *F*(6, 45) = 2.54, *p* = .03. This indicates that even after controlling for the effects of weight gain, a significant difference was observed between AN-R and AN-BP in terms of improvement on the Impulse subscale, *F*(1,50) = 14.14, *p* < .001. Examination of the means indicated that AN-BP improved more during treatment than AN-R, with respect to inhibiting impulsive behaviors when experiencing negative emotions.

### Emotion regulation difficulties and eating psychopathology

To address the third aim of this study, a series of univariate regression models were conducted to assess whether change in each of the DERS subscales were predictors of change in eating disorder psychopathology (i.e., Global EDE-Q). Findings from these analyses indicated that for each DERS subscale, changes during treatment significantly predicted changes in eating disorder psychopathology across time, *p*s < .05, above and beyond the impact of weight gain. Next, a stepwise multiple regression analysis was conducted with all six DERS subscales included as potential predictors. As mentioned, since depression and anxiety scores were not associated with changes in EDE-Q Global score, they were not included as covariates in the following models. Results indicated that only changes in the Goals and Clarity subscales during treatment provided significant predictive contributions to the model, *p*s < .05. Accordingly, only these two subscales were included in a subsequent multiple linear regression model with the DERS Goals and Clarity subscales entered together. Overall, this model significantly predicted changes in eating disorder psychopathology during treatment, *F* (2, 51) =14.29, *p* < .001, *R*^*2 =*^ .36 (See Table 5 for beta weights and other statistics for the individual predictors). This indicates that greater improvements during treatment with respect to emotional clarity and engagement in goal directed behaviours when upset accounted for approximately 36 % of corresponding improvements in eating disorder psychopathology.

**Table 5:** Multiple regression model predicting improvements in EDE-Q global score from improvements in DERS subscales during treatment (*n* = 53)

Model	Predictor	*Β*	*SE*	β	*t*	*p*	*r*
1	DERS-Goals	.08	.03	.34	2.87	.006	.48
	DERS-Clarity	.08	.03	.39	3.25	.002	.51

*Note*. DERS = difficulties in emotion regulation scale

## Discussion

This study examined difficulties with emotion regulation in a sample of patients with AN who were admitted to a specialized intensive hospital-based treatment program. Our first objective was to compare the two AN subtypes in terms of emotion regulation deficits at baseline. As predicted, it was found that patients with AN-BP reported greater difficulties with emotion regulation overall, and in particular with refraining from impulsive behaviors when experiencing negative emotions and accessing adaptive emotion regulation strategies. This is consistent with the findings of Fischer and colleagues [29] who also found that the binge-purge subtype of AN was associated with greater impulse control difficulties but different from those of Haynos and colleagues [31] who found no differences in emotion regulation between subtypes. Contrary to our predictions, we did not find that patients with the AN-R subtype had greater difficulties with emotion awareness or non-acceptance of emotions compared to those with the AN-BP subtype. Instead, these emotion regulation difficulties appear to be equally common in both subtypes. This indicates individuals with both subtypes of AN struggle with a broad range of emotion regulation deficits with respect to understanding, accepting, and effectively managing their emotions. Greater difficulties with impulse control in AN-BP patients may reflect the fact that by definition they are more likely to engage in impulsive and uncontrolled behaviors while under distress (i.e., binge eating, purging) as part of their disorder, whereas individuals with AN-R typically do not. Thus, although both groups experience significant difficulties, these effects were particularly pronounced for patients with AN-BP, suggesting that this group may in fact be particularly impaired.

Our second objective was to examine whether emotion regulation difficulties improved during treatment among those who successfully completed the program. In contrast to the findings of Haynos and colleagues [31], but in line with the findings of Ben-Porath and colleagues [33], and consistent with our own predictions, patients with both AN subtypes made improvements with respect to all facets of emotion regulation from pre-treatment to post-treatment in this study. Although both groups made significant improvements during treatment, AN-BP patients made more pronounced improvements during treatment on impulse control, compared to AN-R patients. This may reflect the fact that AN-BP patients had higher baseline difficulties with impulse control, therefore leaving them with greater room for improvement during treatment. Nevertheless, patients who completed treatment experienced improvements overall in their ability to use strategies to regulate their emotions, to more effectively inhibit impulsive behaviours, and to remain goal directed while under distress, and improved in their awareness of emotions, acceptance of emotions, and ability to differentiate between emotions.

After controlling for the effects of weight gain on pre-to-post treatment changes in emotion regulation difficulties, improvements in emotion regulation skills overall were no longer statistically significant. However, the multivariate interaction remained significant, indicating that even after controlling for the effects of weight regain, the AN-BP group continued to experience greater improvements in impulse control compared to AN-R patients. The fact that pre-to-post treatment improvements in emotion regulation overall were no longer observed after controlling for the effect of weight gain may suggest that weight restoration improves emotion regulation difficulties independent of the effects of psychological therapy. However, this conceptualization is inconsistent with the results of Haynos and colleagues [31] who found no improvement in emotion regulation skills following weight restoration. One possibility for these seemingly inconsistent findings is that weight restoration and emotion regulation skills training may work together to improve emotion regulation skills in individuals with AN. Treatment programs in both our study and Ben-Porath and colleagues’ [33] study involved a substantial focus on psychological interventions including group dialectic behavior therapy (DBT) skills training. This approach is unique from the treatment program in Haynos and colleagues’ [31] study, which did not have an emotion regulation focus. These between-study differences in treatment programs may explain why Haynos and colleagues [31] did not observe emotion regulation improvements following weight restoration, whereas our study and Ben-Porath and colleagues’ study [33] did show that emotion regulation improved following weight restoration. Unfortunately, a limitation of the current study is that we did not have a comparison treatment condition and therefore there is no way of knowing for certain whether the observed changes were due to differences in treatment programs or to other factors such as improved nutrition and weight status. Of note, since EDE-Q and DERS data were not available for patients who dropped out of treatment or were discharged prematurely, these finding only apply to treatment completers.

Our final objective was to examine whether changes in difficulties with emotion regulation predicted changes in eating disorder psychopathology during treatment. Weight gain during treatment was not a significant predictor of improvements in eating disorder psychopathology, so we did not control for the effects of weight gain. It was found that improvements in the ability to differentiate emotions, and the ability to remain goal-directed when experiencing negative emotions predicted improvements in eating disorder psychopathology from pre-treatment to post-treatment. These findings are consistent with recent research by Racine and Wildes [5] who found that emotion regulation deficits predicted AN symptom severity over the year following intensive treatment [30]. It is possible that eating disorder symptoms reduce emotional clarity, and with improvement to eating disorder psychopathology this emotional clarity also improves. Alternatively, it is possible that as individuals learn to identify and differentiate their emotions, they are better able to cope with them rather than engaging in eating disorder behaviours. Additionally, it is likely that during treatment, individuals learn strategies to inhibit urges and remain goal directed even while under distress, which may result in improvements both to emotion regulation and to eating disorder psychopathology. Although it cannot be determined from this study whether improvements in emotion regulation result in improved eating disorder psychopathology or vice versa, or whether in fact the treatment exerts separate but concurrent effects on both sets of problems, this finding demonstrates that changes in both variables are certainly associated.

Taken together, the findings of this study suggest that while weight restoration may play an important role in the treatment of AN, individuals who make more substantial improvements in a range of emotion regulation skills during treatment are also more likely to make improvements to their eating disorder psychopathology, and that this latter relationship appears to occur independently from weight regain. While causal conclusions cannot be drawn about the observed correlational relationships, the findings suggest that recovery from AN may be associated with: 1) an initial focus on weight restoration; and 2) a subsequent emphasis on emotion regulation skills training.

The present findings have a number of potential clinical implications. Some authors have argued that existing treatments for AN have paid insufficient attention to the issue of emotion dysregulation, particularly the ability to tolerate and effectively regulate emotions, and that this may partially explain the lack of effective treatments for adult AN [13, 24]. To date, treatments designed to improve emotion regulation skills, such as DBT, have been tested almost exclusively in eating disorder patients with bulimia nervosa and binge eating disorder [43–45] . Interventions to target emotional over-control typical in AN has received almost no research attention [24]. Our findings suggest that increasing emotion awareness and improving the ability to tolerate distress and engage in adaptive behaviors when distressed is an important part of overcoming AN. Racine and Wildes [30] have found evidence that improvements in emotion regulation are likely to have a positive impact on the longitudinal course of AN. Our results support this hypothesis and suggest that both weight restoration and improvements in emotion regulation are important targets of treatment in AN. In future research, it will be important to examine in randomized controlled studies whether integrating emotion regulation skills training into existing treatments for AN improves treatment outcome.

The current study had a number of limitations. First, we utilized self-report questionnaire measures to assess eating disorder behaviors. Previous research has shown that self-report questionnaires are somewhat less reliable than interview measures of these symptoms [46], suggesting that future research may benefit from a replication of this study employing interview measures of eating disorder behaviors. Second, post-treatment DERS and EDE-Q data were available for treatment completers only. Although there were no baseline differences between completers and non-completers, it is possible that the results might have been different if we had been able to include non-completers in our analyses. Third, this was a correlational study and it is therefore important to be cautious about interpreting the results. Specifically, we cannot conclude that improvements in emotion regulation played a causal role in determining the level of improvement in eating disorder psychopathology. Fourth, there were no follow-up assessments of patients beyond the discharge assessment and we therefore do not know whether the relationships that we observed persisted after treatment. Finally, this study was conducted using a sample of patients with AN attending an inpatient treatment program who are likely to represent more severe cases. It will be important to investigate in future studies whether the observed relationships hold true in a less severely ill sample, such as individuals receiving outpatient treatment in the community.

## Conclusion

In conclusion, findings from the present study add to growing evidence that eating disorder symptoms may serve an emotion regulation function in AN. Specifically, the findings suggest that improvements in emotion regulation skills during treatment are associated with improvements in eating disorder psychopathology, above and beyond the effect of weight restoration on eating disorder psychopathology. Thus, developing interventions to address emotion regulation deficits may be an important component to improving treatment outcome in AN.

## References

[1] Bruch H (1973). Eating disorders: obesity, anorexia nervosa and the person within.

[2] Bruch H (1982). Psychotherapy in anorexia nervosa. Int J Eat Disord.

[3] Engel S, Wonderlich S, Crosby R, Mitchell J, Crow S, Gordon K (2013). The role of affect in the maintenance of anorexia nervosa: evidence from a naturalistic assessment of momentary behaviors and emotion. J Abnorm Psychol.

[4] Harrison A, Sullivan S, Tchanturia K, Treasure J (2010). Emotional functioning in eating disorders: attentional bias, emotion recognition and emotion regulation. Psychol Med.

[5] Racine S, Wildes J (2015). Dynamic longitudinal relations between emotion regulation difficulties and anorexia nervosa symptoms over the year following intensive treatment. J Consult Clin Psychol.

[6] Schmidt U, Treasure J (2006). Anorexia nervosa: valued and visible. A cognitive-interpersonal maintenance model and its implications for research and practice. Br J Clin Psychol.

[7] Treasure J, Schmidt U (2013). DBS for treatment-refractory anorexia nervosa. Lancet.

[8] Corstorphine E (2006). Cognitive-emotional-behavioural therapy for the eating disorders: working with beliefs about emotions. Eur Eat Disord Rev.

[9] Fox J (2009). A qualitative exploration of the perception of emotions in anorexia nervosa: a basic emotion and developmental perspective. Clin Psychol Psychother.

[10] Wildes J, Ringham R, Marcus M (2010). Emotion avoidance in patients with anorexia nervosa: initial test of a functional model. Int J Eat Disord.

[11] Lavender J, Wonderlich S, Engel S, Gordon K, Kaye W, Mitchell J (2015). Dimensions of emotion dysregulation in anorexia nervosa and bulimia nervosa: A conceptual review of the empirical literature. Clin Psychol Rev.

[12] Gratz K, Roemer L (2004). Multidimensional assessment of emotion regulation and dysregulation: development, factor structure, and initial validation of the difficulties in emotion regulation scale. J Psychopathol Behav Assess.

[13] Haynos AF, Fruzzetti AE (2011). Anorexia nervosa as a disorder of emotion dysregulation: evidence and treatment implications. Clin Psychol Sci Pract.

[14] Leon GR, Fulkerson JA, Perry CL, Keel PK, Klump KL (1999). Three to four year prospective evaluation of personality and behavioral risk factors for later disordered eating in adolescent girls and boys. J Youth Adolesc.

[15] Stice E (2002). Risk and maintenance factors for eating pathology: a meta-analytic review. Psychol Bull.

[16] Aldao A, Nolen-Hoeksema S, Schweizer S (2010). Emotion-regulation strategies across psychopathology: a meta-analytic review. Clin Psychol Rev.

[17] Corstorphine E, Mountford V, Tomlinson S, Waller G, Meyer C (2007). Distress tolerance in the eating disorders. Eat Behav.

[18] Troop N, Holbrey A, Trowler R, Treasure J (1994). Ways of coping in women with eating disorders. J Nerv Ment Dis.

[19] Harrison A, Sullivan S, Tchanturia K, Treasure J (2009). Emotion recognition and regulation in anorexia nervosa. Clin Psychol Psychother.

[20] Brockmeyer T, Holtforth M, Bents H, Kämmerer A, Herzog W, Friederich H (2012). Starvation and emotion regulation in anorexia nervosa. Compr Psychiatry.

[21] Brockmeyer T, Skunde M, Wu M, Bresslein E, Rudofsky G, Friederich H (2014). Difficulties in emotion regulation across the spectrum of eating disorders. Compr Psychiatry.

[22] Gilboa-Schechtman E, Avnon L, Zubery E, Jeczmien P (2006). Emotional processing in eating disorders: specific impairment or general distress related deficiency?. Depress Anxiety.

[23] Nowakowski ME, McFarlane T, Cassin S (2013). Alexithymia and eating disorders: a critical review of the literature. J Eat Disord.

[24] Lynch TR, Gray KL, Hempel RJ, Titley M, Chen EY, O’Mahen HA (2013). Radically open-dialectical behavior therapy for adult anorexia nervosa: feasibility and outcomes from an inpatient program. BMC Psychiatry.

[25] Cassin S, von Ranson K (2005). Personality and eating disorders: a decade in review. Clin Psychol Rev.

[26] Shafran R, Cooper Z, Fairburn C (2002). Clinical perfectionism: a cognitive-behavioural analysis. Behav Res Ther.

[27] Geller J, Cockell SJ, Hewitt PL, Goldner EM, Flett GL (2000). Inhibited expression of negative emotions and interpersonal orientation in anorexia nervosa. Int J Eat Disord.

[28] Haynos A, Crosby R, Engel S, Lavender J, Wonderlich S, Le Grange D (2015). Initial test of an emotional avoidance model of restriction in anorexia nervosa using ecological momentary assessment. J Psychiatr Res.

[29] Fischer S, Smith G, Cyders M (2008). Another look at impulsivity: a meta-analytic review comparing specific dispositions to rash action in their relationship to bulimic symptoms. Clin Psychol Rev.

[30] Racine SE, Wildes JE (2013). Emotion dysregulation and symptoms of anorexia nervosa: the unique roles of lack of emotional awareness and impulse control difficulties when upset. Int J Eat Disord.

[31] Haynos AF, Roberto CA, Martinez MA, Attia E, Fruzzetti AE (2014). Emotion regulation difficulties in anorexia nervosa before and after inpatient weight restoration. Int J Eat Disord.

[32] Keys A, Brožek J, Henschel A, Mickelsen O, Taylor H (1950). The biology of human starvation. (2 vols).

[33] Ben-Porath D, Federici A, Wisniewski L, Warren M (2014). Dialectical behavior therapy: does it bring about improvements in affect regulation in individuals with eating disorders?. J Contemp Psychother.

[34] Maxwell S, Lau M, Howard G (2015). Is psychology suffering from a replication crisis? what does ‘failure to replicate’ really mean?. Am Psychol.

[35] Moonsinghe R, Khoury MJ, Janssens ACJW (2007). Most published research findings are false-But a little replication goes a long way. PLoS Med.

[36] Association AP (2000). Diagnostic and statistical manual of mental disorders.

[37] Fairburn CG, Cooper Z (1993). The Eating Disorder Examination. C. G. Fairburn GTW, C. G. Fairburn, G. T. Wilson (Eds).

[38] Fairburn CG, Bèglin SJ (1994). Assessment of eating disorders: interview or self-report questionnaire. Int J Eat Disord.

[39] Luce KH, Crowther JH (1999). The reliability of the eating disorder examination-self-report questionnaire version (EDE-Q). Int J Eat Disord.

[40] Rizvi S, Peterson C, Crow S, Stewart AW (2000). Test-retest reliability of the eating disorder examination. Int J Eat Disord.

[41] Tabachnick BG, Fidell LS (2001). Using multivariate statistics.

[42] Derogatis LR, Melisaratos N (1983). The brief symptom inventory: an introductory report. Psychol Med.

[43] Safer D, Robinson A, Jo B (2010). Outcome from a randomized controlled trial of group therapy for binge eating disorder: comparing dialectical behavior therapy adapted for binge eating to an active comparison group therapy. Behav Ther.

[44] Safer D, Telch C, Agras W (2001). Dialectical behavior therapy for bulimia nervosa. Am J Psychiatry.

[45] Telch C, Agras W, Linehan M (2001). Dialectical behavior therapy for binge eating disorder. J Consult Clin Psychol.

[46] Carter JC, Aime AA, Mills JS (2001). Assessment of bulimia nervosa: a comparison of interview and self-report questionnaire methods. Int J Eat Disord.

